# Design of a novel tryptophan-rich membrane-active antimicrobial peptide from the membrane-proximal region of the HIV glycoprotein, gp41

**DOI:** 10.3762/bjoc.8.130

**Published:** 2012-07-24

**Authors:** Evan F Haney, Leonard T Nguyen, David J Schibli, Hans J Vogel

**Affiliations:** 1 Department of Biological Sciences, University of Calgary, 2500 University Drive NW, Calgary, Alberta, Canada, T2N 1N4

**Keywords:** antimicrobial peptides, cytotoxic peptides, NMR solution structure, membrane interactions, peptides structure–function relationship

## Abstract

A number of physicochemical characteristics have been described which contribute to the biological activity of antimicrobial peptides. This information was used to design a novel antimicrobial peptide sequence by using an intrinsically inactive membrane-associated peptide derived from the HIV glycoprotein, gp41, as a starting scaffold. This peptide corresponds to the tryptophan-rich membrane-proximal region of gp41, which is known to interact at the interfacial region of the viral membrane and adopts a helical structure in the presence of lipids. Three synthetic peptides were designed to increase the net positive charge and amphipathicity of this 19-residue peptide. Ultimately, the peptide with the greatest degree of amphipathicity and largest positive charge proved to be the most potent antimicrobial, and this peptide could be further modified to improve the antimicrobial activity. However, the other two peptides were relatively ineffective antimicrobials and instead proved to be extremely hemolytic. This work demonstrates a novel approach for the design of unexplored antimicrobial peptide sequences but it also reveals that the biological and cytotoxic activities of these polypeptides depend on a number of interrelated factors.

## Introduction

Antimicrobial peptides (AMPs) continue to attract significant attention as potential alternatives to conventional antibiotics. A large number of AMP sequences have been reported in the literature, ranging from diverse peptides isolated from different natural sources to synthetic peptides generated with high-throughput screening methods. From this large sample size, a number of characteristics have been identified that all contribute to the antimicrobial potency of these polypeptides. In this study, we attempted to design a novel AMP using a helical peptide scaffold known to interact at the surface of phospholipid bilayers. Using this template, a series of derivatives were synthesized to augment many of the important antimicrobial factors, with the goal of generating a novel peptide sequence with enhanced antimicrobial potency and weak cytotoxic activity.

The first step in this process was to select an appropriate sequence to serve as the peptide scaffold. Many linear AMPs are unstructured in aqueous solution and only adopt a well-defined structure in the presence of a lipid bilayer [1]. This binding event is integral to the mechanism of action of the peptide, either through direct damage to the phospholipid bilayer or by allowing the peptide to cross the bacterial membrane to reach intracellular targets [2]. A number of AMPs form amphipathic α-helices when bound to lipid bilayers with the hydrophilic residues clustering on one face of the peptide, while hydrophobic residues appear on the opposite face [3]. This amphipathic structure allows the peptide to embed itself into the interfacial region of a phospholipid membrane and it anchors the peptide to the surface of the bilayer.

Another common feature of several AMPs is an unusually high proportion of specific amino acids [4]. Our group has been particularly interested in AMPs rich in tryptophan and cationic amino acids [5–6]. The cationic residues (Arg and Lys) are thought to mediate the initial electrostatic attraction to the negatively charged bacterial cell surface [7]. On the other hand, Trp residues have the unique property that they bind in the interfacial region of a membrane [8–9], thereby anchoring the peptide to the bilayer surface.

Based on these properties, we sought a Trp-rich, membrane-associated region of a protein that does not possess intrinsic antimicrobial activity. Peptides derived from the envelope HIV-1 glycoproteins, gp120 and gp41, have previously been examined for their antimicrobial activity [10] and we chose to use the membrane-proximal region of gp41 as our starting peptide scaffold. This region of the gp41 protein is of particular interest, because it contains five Trp residues, is believed to bind at the surface of the viral membrane [11] and plays an important role in fusing the viral membrane to the target cell membrane. The solution structure of the 19-residue peptide (gp41w) bound to micelles was previously reported by our group [12], and it was shown that this peptide adopts a well-defined helix in the presence of detergent micelles, with four of the five Trp residues distributed in a plane along the length of the peptide. Therefore, this peptide satisfies two of the features that we were looking for, i.e., helical structure and high Trp content. It follows from our current understanding of structure–function relationships of AMPs that adding positive charges and increasing the degree of amphipathicity of this membrane-associated peptide could generate a peptide sequence with potent antimicrobial activity.

**Figure 1:**
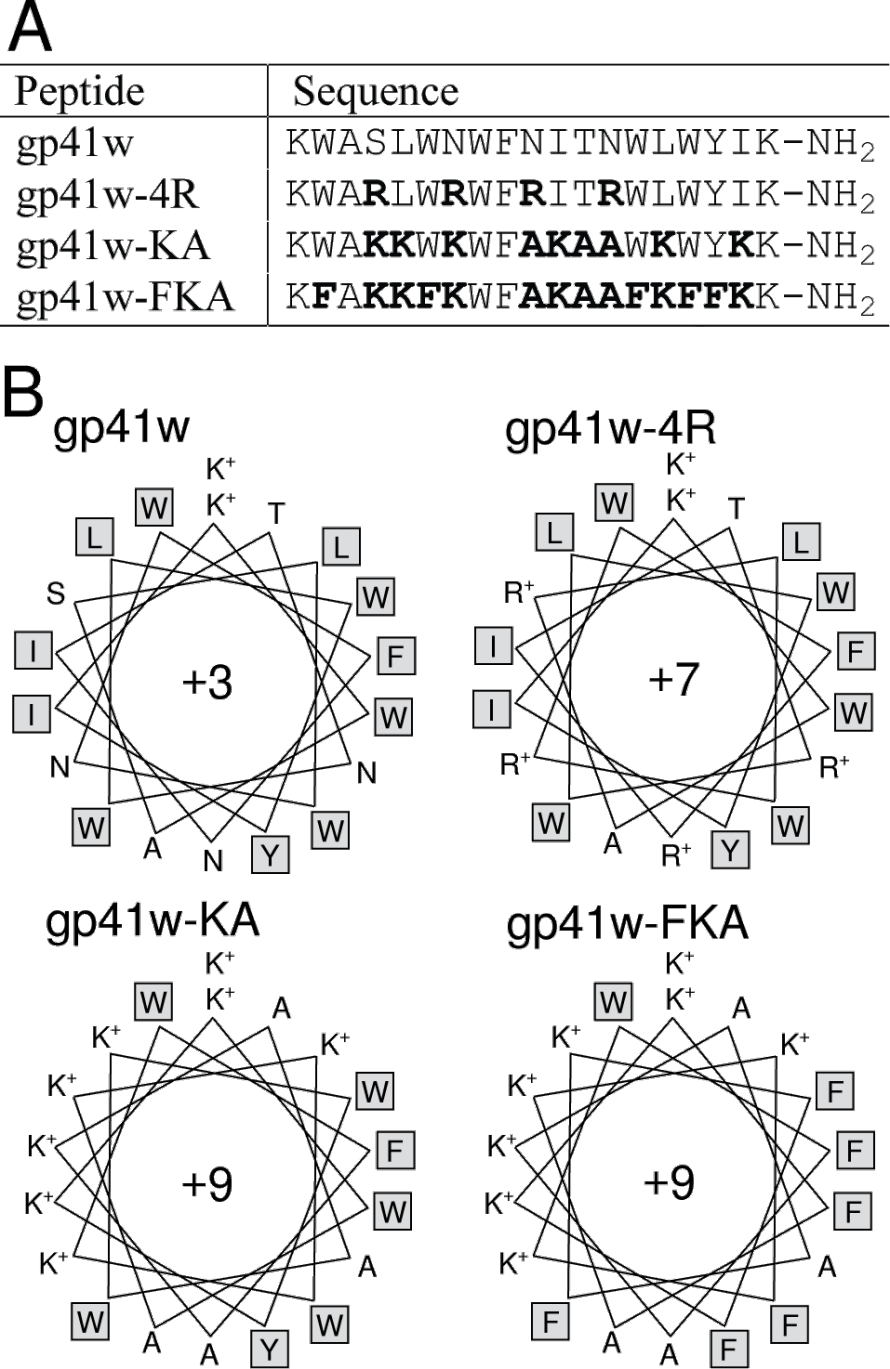
(A) Names and sequences of the gp41w-derived peptides. (B) Helical-wheel projections of gp41w, gp41w-4R, gp41w-KA and gp41w-FKA showing the hydrophobic and large aliphatic side chains in grey boxes. These helical wheels show the amphipathicity of the gp41w derivatives as the hydrophobic residues are increasingly segregated from the hydrophilic and charged residues.

Three derivatives were synthesized based on the amino-acid sequence of gp41w (Figure 1). The first peptide, gp41w-4R, has the polar uncharged amino acids in gp41w replaced with Arg residues. These mutations increased the net charge of the peptide from +3 to +7 and should increase the electrostatic attraction to bacterial cells, which are characterized by a negative surface charge [7]. The second derivative, gp41w-KA, is based on the helical-wheel representation of gp41w and has mutations in the sequence to increase both the net positive charge and amphipathicity of the peptide, while maintaining the relative positions of the bulky hydrophobic residues. Compared to the helical-wheel projection of gp41w-4R, the gp41w-KA peptide removes two of the positive charges from the hydrophobic Trp-rich face of the peptide and replaces them with Ala. In addition, the aliphatic Ile and Leu residues are removed from the hydrophilic surface and are replaced with cationic residues. This generates a peptide with a large net positive charge (+9) and if it can still adopt a helical conformation, then it will be highly amphipathic. The final gp41w derivative, gp41w-FKA, has the same sequence as gp41w-KA except that all of the Trp residues, apart from Trp8, have been replaced by Phe residues. Because the Trp and Phe amino-acid side chains insert in a different manner into membranes, it is interesting to examine the effect of replacing the interface-binding indole groups [13] with the more hydrophobic and deeper penetrating phenyl moieties [8].

## Results

### Antimicrobial and hemolytic activities

The antimicrobial activity for gp41w and its derivatives was determined against Gram-negative *E. coli* and Gram-positive *S. aureus* strains (Table 1). Three of the peptides, gp41w, gp41w-4R and gp41w-KA, were inactive at the concentrations tested. Only gp41w-FKA displayed significant antimicrobial activity against the two bacterial strains, with bacteriostatic and bactericidal effects observed at concentrations ranging from 10–50 μg/mL. The effect of adding gp41w to red blood cells caused minimal hemolysis, indicating that the parent peptide is not particularly cytotoxic. However, the mutation of four residues to cationic Arg residues resulted in a dramatic increase in the hemolytic activity, with significant hemolysis occurring at gp41w-4R concentrations as low as 7 μg/mL. For gp41w-KA and gp41w-FKA, the hemolytic activity was stronger than gp41w, but the effect was not as pronounced as seen with gp41w-4R. Gp41w-KA was hemolytic at a peptide concentration of 42 μg/mL, while gp41w-FKA lysed red blood cells at a concentration of 195 μg/mL. In the case of gp41w-FKA, the hemolytic concentration was substantially higher than the minimum inhibitory concentration (MIC) and minimum bactericidal concentration (MBC) values (Table 1).

**Table 1:** Antimicrobial and hemolytic activities of the gp41w-derived peptides. All concentrations are presented as μg/mL.

Peptide	MIC^a^ *E. coli*	MBC^b^ *E. coli*	MIC^a^ *S. aureus*	MBC^b^ *S. aureu*s	Hemolysis EC_50_^c^

gp41w	>100	>100	>100	>100	550
gp41w-4R	>100	>100	>100	>100	7–14
gp41w-KA	>100	>100	>100	>100	42
gp41w-FKA	10–30	40–50	<10	20–30	195

^a^Minimum inhibitory concentrations (MIC); ^b^minimum bactericidal concentrations (MBC); ^c^effective concentration for 50% hemolysis.

### Tryptophan fluorescence spectroscopy

**Figure 2:**
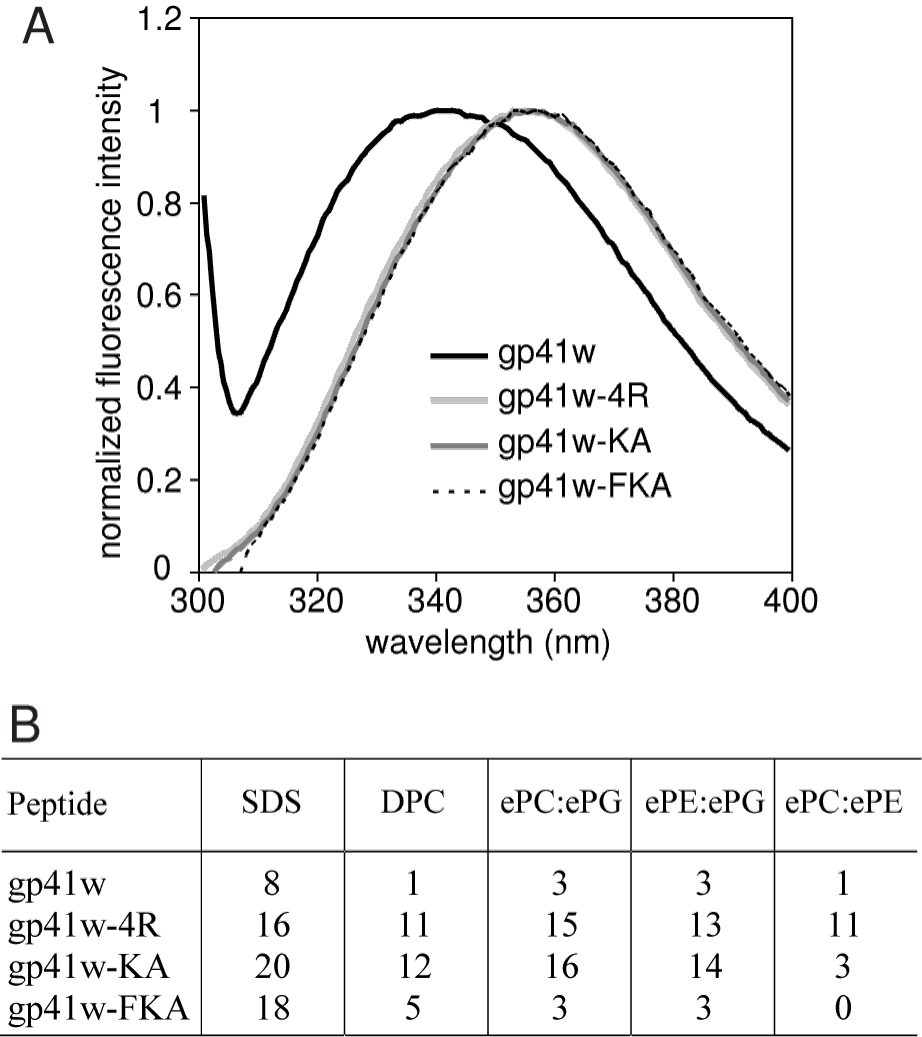
(A) Normalized Trp emission spectra of the gp41w derivatives in buffer. The spectra have been normalized to the fluorescence intensity value measured at the wavelength of the emission maximum. (B) Blue shifts (nm) in the Trp maximum-emission wavelength for gp41w, gp41w-4R, gp41w-KA and gp41w-FKA in the presence of micelles and large unilamellar vesicles (LUVs) of varying composition. All wavelengths of the maxima are based on average values from three separate scans. Abbreviations for the detergents and lipids are: SDS, sodium dodecylsulfate; DPC, dodecylphosphocholine; ePC, egg-derived L-α-phosphatidylcholine; ePE, egg-derived L-α-phosphatidylethanolamine; ePG, egg-derived L-α-phosphatidylglycerol; Chol, cholesterol.

Tryptophan emission fluorescence of all the gp41w derivatives was performed to examine the microenvironment surrounding the Trp side chains. The emission spectra of the four peptides in aqueous buffer are shown in Figure 2. It is evident that the behaviour of the gp41w peptide is very different from the other peptides. The wavelength of the maximum emission for gp41w occurs at 342 nm, while the other three peptides have maximum emission wavelengths at ~356 nm. The value of 356 nm is normal for an exposed Trp side chain in aqueous solution. Apparently, the environments surrounding the Trp residues of the parent gp41w are significantly different from the three derivative peptides, resulting in a blue shift in the wavelength of the emission maximum. A shift towards lower wavelengths is usually interpreted as the Trp residue residing in a more hydrophobic environment, which may be due to the gp41w peptides oligomerizing in solution.

The addition of detergent micelles and large unilamellar vesicles (LUVs) to the peptide solutions caused substantial blue shifts of the maximum wavelength in the Trp emission spectra (Figure 2B). The maximum wavelength of the gp41w-4R samples blue shifted in all the lipid environments, with the largest changes occurring in the presence of anionic LUVs and detergents. Gp41w-KA also displayed large blue shifts in the presence of negatively charged lipid species, while only small blue shifts were seen with zwitterionic ePC:ePE LUVs. Gp41w-FKA displayed similar behaviour to gp41w-KA except that the blue shifts were not as pronounced for the negatively charged lipids and there was almost no blue shift seen when the peptide was added to neutral LUVs. The helical-wheel projection of gp41w-FKA places Trp8 in the hydrophilic face of the helix (Figure 1) and the NMR structure, determined in the cosolvent mixture of chloroform, methanol and water, places the indole side chain at the interface of the charged and hydrophobic sides of the helix (see below). Therefore, Trp8 likely does not penetrate as deeply into the acyl chains of the vesicles. The blue shifts seen in the gp41w samples were also comparatively small, but this is likely due to the unusual emission maximum measured for this particular peptide in aqueous buffer. Therefore, the smaller blue shifts for gp41w can be attributed to the peptide dissociating from the oligomeric form followed by peptide insertion into the bilayer.

Acrylamide quenching experiments were performed for the four peptides to examine the accessibility of the Trp fluorophores in the various membrane environments. If a Trp side chain inserts into the hydrophobic core of the bilayer, it becomes less accessible to the effects of the soluble acrylamide quencher. The quenching of Trp fluorescence is directly related to the concentration of quencher, so a titration with the neutral acrylamide allows for the calculation of the Stern–Volmer constant (*K*_sv_), which quantitatively measures the accessibility of the Trp residues in various lipid environments.

**Figure 3:**
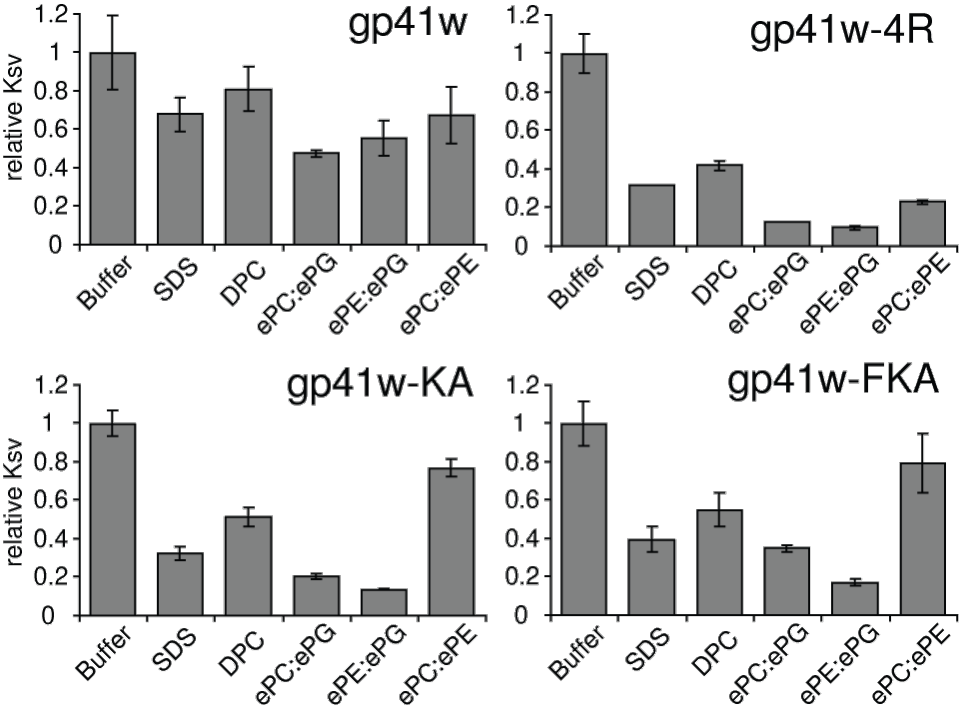
Relative *K*_sv_ values calculated for gp41w, gp41w-4R, gp41w-KA and gp41w-FKA in buffer and in the presence of detergent micelles and phospholipid LUVs. Error bars represent the standard deviation of three separate trials. Values are reported as relative values compared to the *K*_sv_ determined in buffer. The numerical values of the buffer *K*_sv_s were 10.3, 27.8, 16.4 and 17.9 for gp41w, gp41w-4R, gp41w-KA and gp41w-FKA, respectively.

The *K*_sv_ values for the gp41w peptides are summarized in Figure 3. Consistent with the other fluorescence results, the *K*_sv_ for gp41w in buffer is significantly smaller than the *K*_sv_ values calculated for the other peptides under the same conditions. This suggests that the Trp residues in gp41w are not as accessible to the acrylamide molecules and further supports the idea that this peptide is present in an oligomeric state in aqueous solution. When detergent micelles or LUVs were added to the gp41w peptide, all of the calculated *K*_sv_ were lower than the values determined in buffer, indicating that gp41w binds to both micelles and LUVs with the Trp residues inserting into the membrane.

All of the gp41w derivatives have large *K*_sv_ values in buffer, which is expected if the Trp fluorophores are exposed to the aqueous buffer. In the presence of detergent micelles and LUVs, all of the calculated *K*_sv_ values for gp41w-4R decreased dramatically, consistent with this peptide binding to the bilayer and the Trp side chains burying into the membrane. This effect was most pronounced in the presence of anionic liposomes, but all of the mixtures resulted in strong decreases in the calculated *K*_sv_ values (Figure 3).

The other two peptides, gp41w-KA and gp41w-FKA, demonstrate a preference for anionic lipids and detergents, as the *K*_sv_ values determined in the presence of SDS micelles and LUVs containing ePG lipids are much lower than those determined in the presence of DPC or zwitterionic liposomes (Figure 3). When the peptides were added to ePC:ePE LUVs, there was only a small decrease in the calculated *K*_sv_. This suggests that these two peptides interact only weakly with the neutral liposomes. The decrease in *K*_sv_ observed in the presence of DPC micelles is likely due to the high concentration of detergent used in these samples. These conditions shift the equilibrium of the free-floating peptide in solution to the micelle-bound form, resulting in the insertion of the Trp side chain into the hydrophobic core of the micelle.

### Calcein leakage

**Figure 4:**
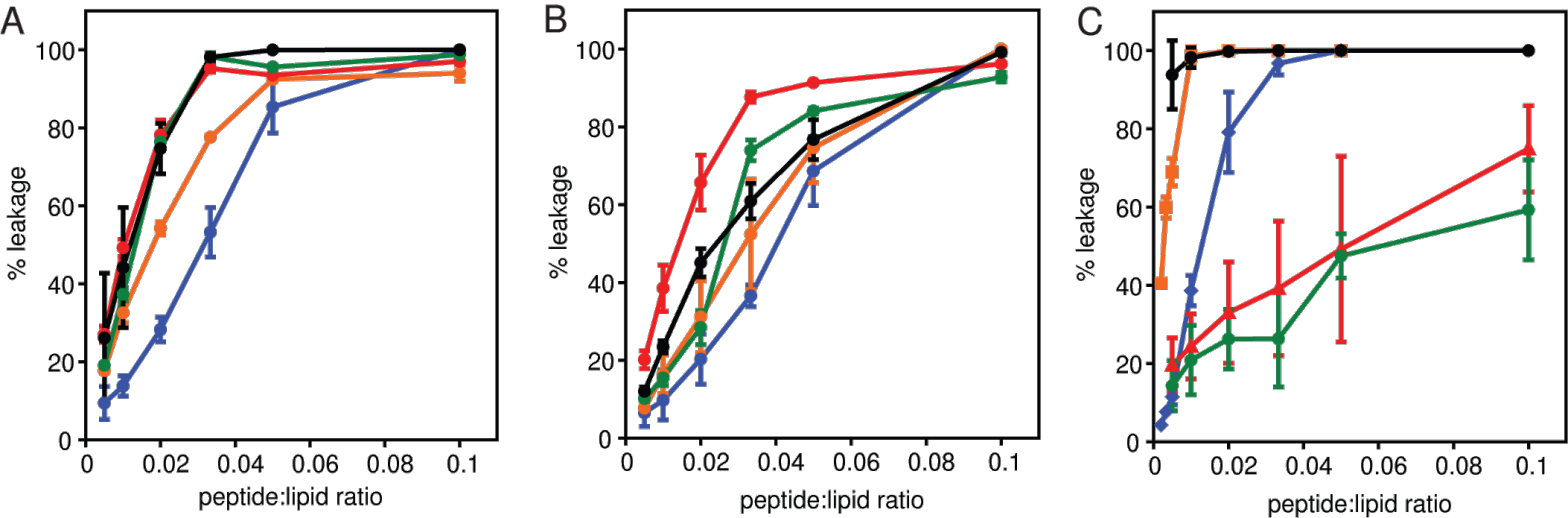
Percent calcein leakage induced by the gp41 derivatives. Gp41w (blue), gp41w-4R (orange), gp41w-KA (red) and gp41w-FKA (green) were added to calcein encapsulated LUVs composed of (A) ePC:ePG, (B) ePE:ePG and (C) ePC:ePE at different peptide:lipid ratios. Melittin (black), a known lytic peptide from honey-bee venom was used as a positive control. All experiments were carried out in triplicate at 37 °C.

All four peptides were tested for their ability to induce the release of the self-quenching fluorescent dye, calcein, from calcein-encapsulated LUVs [14–15]. All of the peptides caused calcein release from negatively charged LUVs composed of ePC:ePG or ePE:ePG, and the percentage of calcein released was similar to that induced by melittin, a known lytic peptide [16]. Interestingly, the amount of leakage induced by each of the gp41 peptides was roughly equivalent, indicating that all of these derivatives disrupt negatively charged liposomes to the same extent (Figure 4A and Figure 4B). When the peptides were added to zwitterionic LUVs composed of ePC:ePE lipids, there was a larger difference in the amount of calcein released from the LUVs. Gp41w-4R caused significant leakage, similar to the effect of melittin, while the gp41w peptide also caused leakage, but not to the same extent. Gp41w-KA and gp41w-FKA caused membrane disruption of the zwitterionic LUVs, but only ~70% leakage was observed at the highest peptide:lipid ratio tested. This demonstrates that these two peptides do not disrupt bilayer organization in neutral liposomes to the same extent as gp41w or gp41w-4R (Figure 4C).

### Differential scanning calorimetry

The gp41w derivatives were added to 1,2-dipalmitoyl-*sn*-glycero-3-phosphocholine (DPPC) and 1,2-dipalmitoyl-*sn*-glycero-3-phospho-(1'-*rac*-glycerol) (DPPG) lipid suspensions and the resulting lipid and peptide mixtures were examined by differential scanning calorimetry (DSC) to determine the effect of the peptides on the thermotropic phase behaviour of phospholipids. Eukaryotic membranes typically have a high phosphatidylcholine content, while bacterial membranes are characterized by phosphatidylethanolamine and anionic phosphatidylglycerol head groups [7]. Therefore, the DPPC lipids serve as a simple model for a eukaryotic membrane, while the anionic DPPG lipids are representative of the negatively charged bacterial membrane.

**Figure 5:**
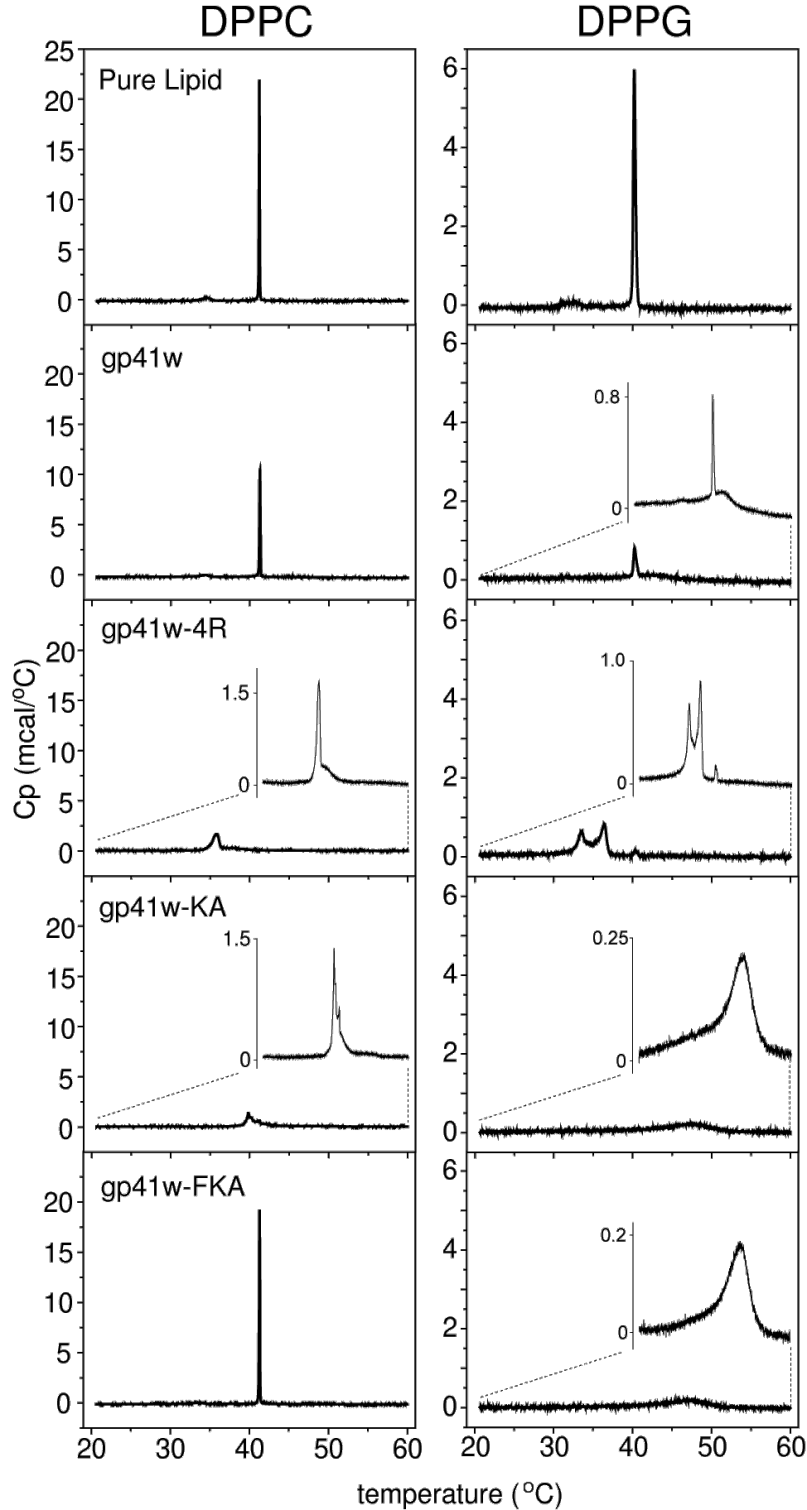
DSC thermograms of pure zwitterionic DPPC (left) and anionic DPPG (right) lipid suspensions compared to lipids mixed with gp41w, gp41w-4R, gp41w-KA and gp41w-FKA. A peptide-to-lipid molar ratio of 1:10 was maintained in all of the samples. The insets are a magnification of the thermogram to show the distribution and shape of the peaks.

When added to DPPC lipid suspensions, gp41w and gp41w-FKA had very little effect on the phase transitions of DPPC lipids (Figure 5). The main phase transition of DPPC, seen at 41 °C, is unaffected by the addition of peptide, while the pretransition at ~34 °C is slightly broadened and shifts to a lower temperature. When gp41w-4R and gp41w-KA were added to the DPPC lipid suspensions, the resulting thermograms showed that these peptides had very drastic effects on the organization of DPPC bilayers (Figure 5). The final heating scan of gp41w-4R mixed with DPPC phospholipids shows a sharp transition at ~36 °C with a broad shoulder between 37–40 °C. This large temperature shift compared to the main phase transition of DPPC indicates that the gp41w-4R peptide destabilizes zwitterionic bilayers more effectively than any of the other gp41w derivatives. For gp41w-KA, there are a number of peaks at temperatures below the phase-transition temperature of pure DPPC, including a sharp transition at ~40 °C, suggesting that this peptide also destabilizes the transition of DPPC from the lamellar-gel to the liquid-crystalline phase. The ability of the gp41w derivatives to disrupt DPPC bilayers correlates with the observed hemolytic activity, and this may explain why gp41w-4R and gp41w-KA readily lyse red blood cells in vitro.

The DSC results of the gp41w derivatives mixed with DPPG lipids demonstrate that all the peptides induce changes in the thermotropic phase behaviour of negatively charged lipids. The pretransition at ~33 °C seen in the DSC trace of pure DPPG lipids disappears in the presence of all the gp41w peptides. For gp41w added to DPPG lipid suspensions, the peak at 40 °C indicates that there is some unbound DPPG that behaves like the pure lipid suspension. However, the emergence of a broad transition at ~43 °C is likely due to the formation of peptide–lipid aggregates. The addition of gp41w-4R to DPPG yields peaks in the thermogram at 33 and 36 °C, suggesting that when gp41w-4R binds to negatively charged liposomes, it destabilizes the organization of the bilayer. Apparently, some unbound DPPG remains as well because there is still a small peak at ~40 °C. The addition of gp41w-KA and gp41w-FKA to DPPG gave remarkably similar DSC results. The main phase transition at 40 °C is abolished in the presence of either peptide and is replaced by a broad transition centered at 47 °C. This could be due to the formation of peptide–lipid aggregates that produce the high-temperature transitions in the thermogram. Interestingly, the degree to which the gp41w derivatives disrupt DPPG lipids does not correlate with the observed antimicrobial activity, suggesting that interaction with negatively charged lipids is not sufficient to explain the observed antimicrobial effects.

### Circular dichroism spectroscopy

**Figure 6:**
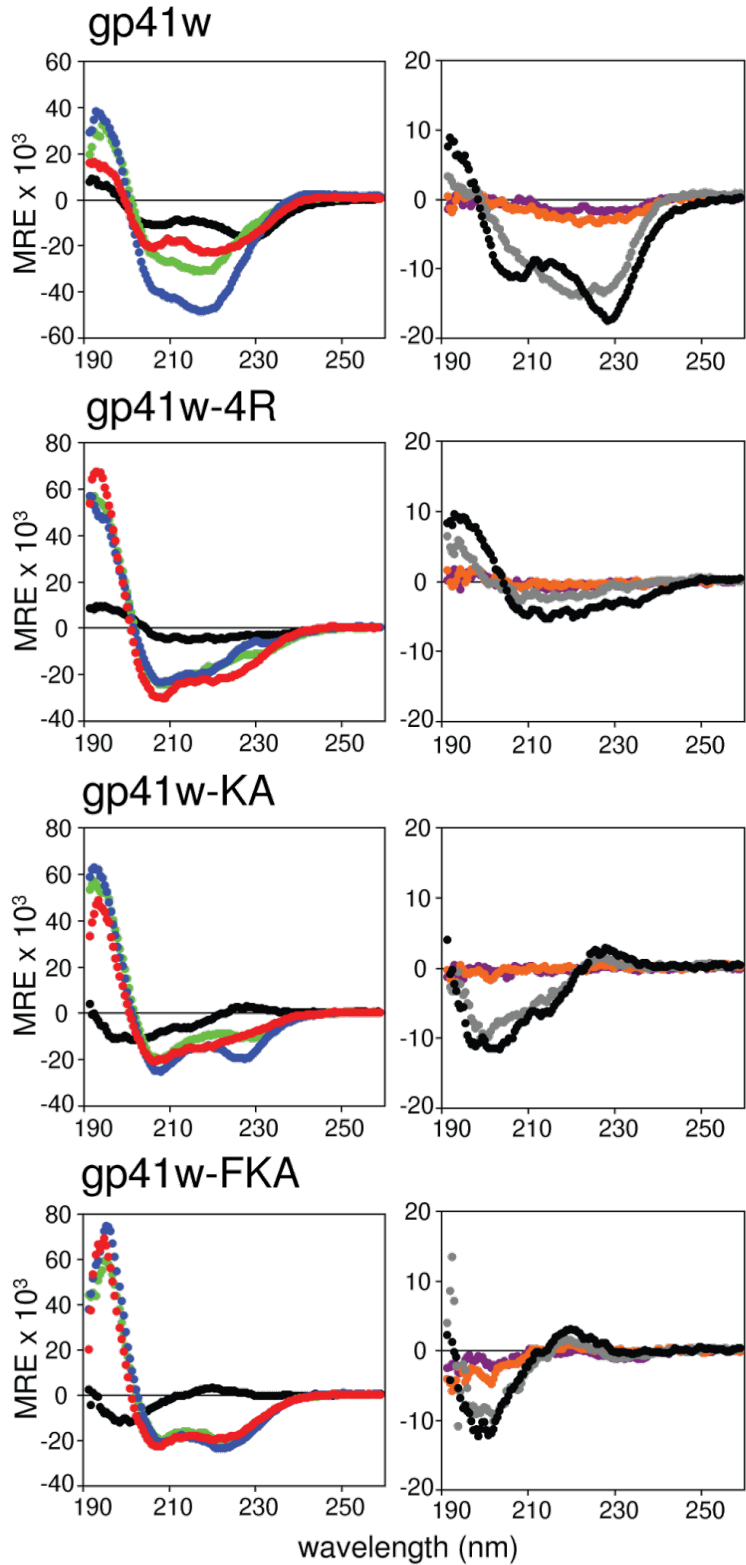
Far-UV CD spectra of gp41w and the three derivative peptides. The panel on the left shows the peptides in phosphate buffer (black), 25 mM SDS (green), 25 mM DPC (yellow) and 50% TFE (red). The panel on the right is the peptides in the presence of LUVs composed of ePC:ePG (violet), ePE:ePG (orange) or ePC:ePE (gray). All data are reported in units of mean residue ellipticity (MRE) with units of deg cm^2^ dmol^−1^ res^−1^.

The backbone conformations of all of the gp41 peptide derivatives were examined by using far-UV circular dichroism (CD) spectroscopy. The resulting CD spectra of the peptides in buffer, SDS, DPC and 50% trifluoroethanol (TFE) are shown in Figure 6. Two of the peptides, gp41w and gp41w-4R, generated unique CD spectra for AMPs dissolved in aqueous solution. Typically, linear AMPs are unstructured in aqueous buffer, resulting in CD spectra with a characteristic minimum at ~200 nm. For gp41w-4R, there is a consistent signal from 235 to 205 nm and a maximum peak at ~195 nm, suggesting that this peptide already adopts a partially helical conformation in aqueous buffer. The CD spectra of gp41w are even more intriguing as there are strong minima observed at 230 and 208 nm. The peak at 208 nm is likely due to the presence of α-helical structure, while the band at ~230 nm can be attributed to stacking interactions between nearby aromatic rings [17]. This is in agreement with the fluorescence results and suggests that the gp41w backbone adopts a regular secondary structure when oligomerized in solution.

The addition of SDS or DPC micelles to gp41w caused a conformational change in the peptide, likely due to the peptide multimers disassociating followed by peptide binding to the micelles. When micelles were mixed with gp41w-4R, an increase in the mean residue ellipticity at 208 and 222 nm was observed, consistent with the formation of an α-helix. When compared to the curve of gp41w-4R in 50% TFE, the traces from the micelle-containing samples show little difference, indicating that the conformation of gp41w-4R is similar in all these environments. In the presence of 50% TFE, gp41w also adopts a largely α-helical conformation, but in the presence of micelles there may be other structural elements that contribute to the CD spectra, as evidenced by the strong minima at ~218 nm seen in both the SDS and DPC traces. However, the micelle samples still have minima at 208 nm and maximum values at 195 nm, both of which are characteristic of helical structure. Therefore, it appears that a large portion of gp41w forms a helix when bound to SDS and DPC micelles.

The CD spectra for gp41w-KA and gp41w-FKA are more typical for AMPs. Both peptides are unstructured in aqueous solution, with strong minima at 200 nm. The addition of micelles or TFE induces a conformational change in the peptides consistent with the formation of helical structure. The micelle-bound conformation of gp41w-KA has another minimum at 227 nm, which may be due to interactions among the Trp side chains [18]. The fact that the CD spectra of gp41w-FKA in the presence of SDS, DPC and 50% TFE overlap almost perfectly with each other suggests that this peptide adopts equivalent structures in all three environments.

The conformations of the gp41w derivatives in the presence of LUVs of varying composition were also examined with CD spectroscopy (Figure 6). In the presence of zwitterionic ePC:ePE LUVs, a slight conformational change was observed for the gp41w peptide while the intensity of the CD signal from gp41w-4R decreased significantly. In the case of gp41w, this is likely due to a minor conformational change as the peptide reorients itself to interact with the LUVs. There was evidence of lipid aggregation in the gp41w-4R sample; therefore the spectral change likely arises from the formation of peptide–lipid aggregates, which no longer absorb the circularly polarized light. Only minor changes were seen in the CD values measured for gp41w-KA and gp41w-FKA compared to the spectra obtained in buffer, suggesting that these two peptides do not readily interact with neutral liposomes.

Peptide–lipid aggregates were observed in all the CD samples containing anionic LUVs composed of ePC:ePG or ePE:ePG, and the intensity of the measured CD signals virtually disappeared for all the peptide samples (Figure 6). This was accompanied by a corresponding decrease in the absorbance values measured by the spectropolarimeter (data not shown). In addition, precipitates could be seen in the cuvette, suggesting that all the gp41w peptides interact strongly with negatively charged lipid species leading to the formation of insoluble peptide–lipid complexes. Unfortunately, due to the loss of CD signal in these samples, it is impossible to determine what type of secondary structure is present in these aggregates.

### NMR solution structure

All the gp41w derivatives were examined by nuclear magnetic resonance (NMR) spectroscopy to determine the high-resolution structure in a membrane mimetic environment. The structure of gp41w was previously determined in the presence of DPC micelles [12]. However, when the three derivatives were tested with SDS and DPC micelles, the resulting NMR spectra were poorly resolved and not suitable for structural analysis (data not shown). Consequently, we chose to use a cosolvent mixture consisting of four parts deuterated chloroform, four parts deuterated methanol and one part water [19–20]. The 2D ^1^H NOESY spectra obtained in this solvent mixture were very well resolved, allowing for identification of all of the backbone atoms and virtually all the side-chain atoms in gp41w-4R, gp41w-KA and gp41w-FKA (Supporting Information File 1, Figure S1). The nuclear Overhauser effect (NOE) patterns of the connections between protons in the gp41w-derived peptides are highly indicative of helical structure (Supporting Information File 1, Figure S2). Interestingly, when gp41w was added to the cosolvent mixture, the peptide did not readily dissolve and there was evidence of aggregation. These aggregates persisted at higher temperatures and the NMR spectra acquired with these samples were of poor quality. As a result, the solution structure of gp41w was not determined in the cosolvent mixture. Structural statistics from the ARIA calculations are shown in the Supporting Information File 1, Table S1.

**Figure 7:**
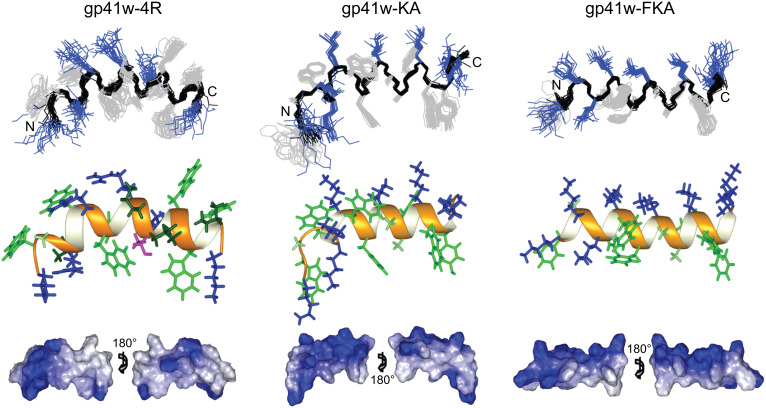
NMR solution structure of gp41w-4R, gp41w-KA and gp41w-FKA in the cosolvent mixture. Overlay of the 20 lowest-energy structures reveals a well-defined backbone conformation for all three peptides (top panel). All the peptides adopt a largely helical conformation as seen in the ribbon diagrams (middle panel). The hydrophobic residues (green) and the cationic residues (blue) are shown along with Thr12 (magenta) in gp41w-4R. The gp41w-4R peptide lacks distinct amphipathicity, while gp41w-KA and gp41w-FKA have much more pronounced amphipathic character across the length of the helix (bottom panel, positively charged regions are indicated in blue).

As expected, similar to gp41w [12], the solution structures of gp41w-4R, gp41w-KA and gp41w-FKA were found to be largely helical, but there are significant differences in the charge distribution and amphipathicity of these peptides, which must be related to the observed biological effects. The solution structure of gp41w-4R forms a well-defined helix across the length of the peptide with a backbone root-mean-square deviation (RMSD) of 0.630 Å (Figure 7). It appears that the structure is slightly bent, especially in the N-terminal region, which lies well below the plane of the helix formed by residues 7–19. The side-chain distribution of gp41w-4R in the cosolvent solution shows that the peptide does not form a distinct amphipathic structure. The hydrophobic side chains and the charged Arg and Lys residues are relatively evenly distributed across the surface of the peptide.

The structure of gp41w-KA in the cosolvent mixture also forms a very well defined helix with a backbone RMSD of 0.389 Å (Figure 7). In agreement with the structure of gp41w-4R, the N-terminal region of gp41w-KA is not aligned with the C-terminal helix formed across residues 7–19 and these first six residues appear to form a slightly extended conformation. This may be an important structural feature of this peptide but could also be due to a low number of intramolecular contacts between the side-chain protons in this region of the peptide. Different from the structure of gp41w-4R, the well-defined helical region of gp41w-KA is largely amphipathic, with the positively charged Lys residues at positions 7, 11, 15, 18 and 19 all appearing on the same face of the peptide, while the opposite face has many of the large hydrophobic residues, including Phe9, Trp16 and Tyr17. In addition, the side chains of Trp6, Trp8 and Trp14 are all found in the same plane of the peptide, which separates the charged Lys surface from the hydrophobic face. The amphipathic structure of gp41w-KA is highly suggestive of a membrane-bound orientation in which the hydrophobic face inserts into the hydrophobic core of the bilayer while the positively charged residues interact with the charged lipid head groups. Based on this model, the N-terminal region of gp41w-KA would then be oriented into the bilayer, which would bring the Lys residues at positions 1, 4 and 5 into the hydrophobic core of the bilayer. This may be related to the membrane-destabilizing properties of the gp41w-KA peptide.

The gp41w-FKA peptide has a similar primary sequence to the gp41w-KA except that all of the Trp residues in gp41w-KA (except Trp8) have been mutated to Phe residues. These changes did not affect the ability of the gp41w-FKA peptide to adopt a helical conformation in the cosolvent mixture of chloroform, methanol and water. The peptide formed a very well defined α-helix across the length of the peptide with a backbone RMSD of 0.426 Å (Figure 7). The side-chain distribution of gp41w-FKA creates the most amphipathic structure of any of the gp41w derivatives. Only Lys1 and Lys5 are not part of the positively charged face formed by the remaining Lys residues. Most of the hydrophobic Phe residues are found opposite the charged face, with the side chain of Trp8 once again located equidistant from the charged surface and the hydrophobic face. A closer examination of the surface-charge distribution of this peptide reveals an uninterrupted hydrophobic face stretching from Phe1 to Phe17, suggesting that gp41w-FKA binds at the interfacial region of a phospholipid bilayer.

## Discussion

AMPs are often considered as potential therapeutic agents because of their selectivity towards bacterial cells and the perceived difficulty associated with bacteria in developing resistance to these molecules [21]. Using an inactive membrane-associated Trp-rich peptide as a scaffold, we attempted to generate a novel AMP based on some of the simple characteristics that have been outlined for AMPs, such as hydrophobicity, amphipathicity, net charge, helical structure and phospholipid bilayer interactions [22]. The results obtained with the gp41w-derived peptides reveal a complex relationship between the structural factors that contribute to the antibacterial activity and the features that determine the hemolytic activity.

A large proportion of AMPs are unstructured in solution, and they adopt amphipathic α-helices when bound to a phospholipid bilayer [3,23]. This binding to the bacterial membrane is often related to a membrane-destabilizing mode of action, which ultimately leads to bacterial cell death [24]. However, recent evidence suggests that membrane binding may be a part of a more complex mode of action involving multiple targets of inhibition [21,25]. In addition to their membrane-binding properties, many AMPs are typified by a high proportion of Trp and cationic amino acids [5–6]. The Trp residues are unique because of their preference for the interfacial region of biological membranes [8–9], while the cationic residues are responsible for the initial electrostatic attraction to the negatively charged bacterial cell [7,22]. In this work, the membrane proximal region of gp41 was chosen as a starting point from which to design a novel AMP sequence. This 19-residue peptide adopts a helical structure with partial amphipathic character in the presence of DPC micelles [12] and contains five Trp residues, four of which form a plane along the helix axis. A model for the membrane interaction of gp41w was proposed wherein the peptide inserts into the interfacial region of the outer leaflet of the viral membrane and this Trp plane resides at the lipid–water interface. The net positive charge of the native gp41w peptide is relatively small (+3). Three peptide derivatives were synthesized to increase the overall charge of the peptide as well as enhance the amphipathicity of the helix with the ultimate goal of generating a novel AMP sequence.

It was expected that the gp41w peptide would have weak antimicrobial and hemolytic activity. Indeed this was the case as no antimicrobial activity was observed against *E. coli* and *S. aureus* at gp41w concentrations below 100 μg/mL, and hemolysis was not observed at peptide concentrations lower than 500 μg/mL. The fluorescence spectroscopy results demonstrate that gp41w interacts with lipid bilayers and the Trp residues embed themselves into the membrane. The DSC data indicate that gp41w interacts with both DPPC and DPPG lipids, since the shape of the main phase-transition peak is significantly different compared to the pure lipid. However, it appears that gp41w does not dramatically alter the bilayer organization, since the melting temperature of the main phase transition remains unchanged. The calcein leakage results suggest that gp41w is membrane active, since it causes leakage of vesicle contents, but it appears that vesicle leakage does not correlate with antimicrobial activity. It could be related to the relatively low positive charge of gp41w, but this seems unlikely since gp41w-4R has four additional positive charges and is also antimicrobially inactive.

The lack of antimicrobial activity may be related to the fact that the gp41w peptide appears to oligomerize in solution. When found in the HIV-1 Env glycoprotein complex, gp41 is known to exist as a trimer [26], and previous attempts to resolve the NMR structure of gp41w in aqueous solution or 40% TFE were unsuccessful because of peptide aggregation or poor solubility [12]. In terms of the antimicrobial activity, gp41w oligomers may be too large to pass through the peptidoglycan layer surrounding Gram-positive bacteria. Additionally, they would have to disassociate into monomers before they could insert themselves into the bacterial membrane. Recent artificial-neural-network prediction models of antimicrobial peptides have found that peptide aggregation in solution indeed contributes to a low antimicrobial activity [27]. Interestingly, the addition of cationic residues to peptides that tend to aggregate in solution has been shown to inhibit aggregation while improving the antimicrobial potency at the same time [28].

The gp41w-4R derivative of the gp41w peptide has the three Asn residues and Ser8 mutated to cationic Arg residues. Arginine residues seem to be preferred over Lys residues in short Trp- and Arg-rich AMPs obtained through combinatorial chemistry [29]. Interestingly, the gp41w-4R peptide did not show any increased antimicrobial activity despite the high content of Trp and cationic residues. Instead, the gp41w-4R peptide unexpectedly demonstrated extremely high hemolytic activity. The solution structure of gp41w-4R in the cosolvent mixture is largely α-helical, but the peptide is not particularly amphipathic. This is consistent with the helical wheel projection of gp41w-4R (Figure 1), which predicted that the positively charged residues would be evenly distributed around the circumference of the helix axis.

The biophysical experiments reveal that gp41w-4R readily interacts with membranes, irrespective of the composition of the head groups in the phospholipids. The effect of gp41w-4R on zwitterionic liposomes is substantial in comparison to the other peptides, especially in the calcein leakage and DSC results, and this behaviour is likely directly related to the strong hemolytic activity of this peptide. The positively charged Arg and Lys side chains in gp41w-4R would prefer to reside in an aqueous environment; however, gp41w-4R also possesses a large number of hydrophobic amino acids, which readily insert into the interfacial region of the bilayer and into the hydrophobic acyl chains [8,30]. Therefore, if the hydrophobic residues penetrate into the hydrophobic core of a membrane, this would also introduce positive charges into the bilayer, thereby destabilizing the membrane. A model such as this can explain the hemolytic activity of gp41w-4R, but the lack of antimicrobial activity suggests that the gp41w-4R peptide does not reach the bacterial cytoplasmic membrane in the presence of bacterial cells.

Amphipathicity is often cited as an important factor that contributes to the effectiveness of AMPs [3–4,23,31–33] and this may explain why the gp41w-4R peptide has poor antimicrobial activity since it is not overly amphipathic. Additionally, the CD spectra of gp41w-4R in buffer suggests that this peptide is somewhat structured in aqueous solution, so a conformational rearrangement of gp41w-4R may be required for membrane destabilization of bacterial cells.

The gp41w-KA and gp41w-FKA peptides are significantly different in primary sequence compared to gp41w, but the common feature of these peptides is the positioning of the bulky hydrophobic residues. The positively charged residues in gp41w-KA and gp41w-FKA were engineered to appear on one side of the helix axis to improve the overall amphipathicity of the α-helix. Indeed, the NMR solution structures of gp41w-KA and gp41w-FKA were found to be largely amphipathic (Figure 7). The C-terminal region in both peptides adopts a well-defined helical structure and only the N-terminal residues appear to deviate from this conformation. Interestingly, the increased amphipathicity appears to be inversely related to the hemolytic activity of these peptides and the interactions with zwitterionic ePC:ePE LUVs or DPPC lipids suspensions are not as substantial as the effects seen with gp41w-4R. In addition, the selectivity for negatively charged membranes over zwitterionic lipids is enhanced in these two peptides, particularly for gp41w-FKA.

The stronger antimicrobial potency of the gp41w-FKA peptide compared to gp41w-KA was unexpected because Trp residues have long been implicated as being important for the activity of AMPs [5–6]. While Trp residues preferentially sit at the interfacial region of a membrane [8–9] and Phe residues show a preference for the interior of the membrane [8,30], our group previously observed an increase in the antimicrobial activity of tritrpticin analogues, wherein the three Trp residues were mutated to Phe [34]. This was accompanied by a decrease in hemolytic concentration of tritrpticin as well. Conversely, mutating Trp residues to Phe residues decreased the antimicrobial potency of indolicidin [35] while increasing the hemolytic activity of this peptide.

At this point, it is unclear why gp41w-FKA is a more potent antibacterial than gp41w-KA. The fluorescence results demonstrate that both peptides preferentially interact with anionic lipids and the calcein leakage profiles of both peptides are similar in the presence of anionic and neutral liposomes. The DSC traces of DPPG mixed with both peptides are virtually identical and the only large difference between the two peptides is that gp41w-KA has a strong effect on DPPC lipid suspensions, which only explains the stronger hemolytic effect of this peptide. Evidently, the antimicrobial mechanism of action of gp41w-FKA involves more than a simple disruption of the bacterial cytoplasmic membrane. Alternatively, gp41w-KA and gp41w-4R may not reach the bacterial cytoplasmic membrane through some unknown interactions with molecules on the surface of the bacterial cell.

Other de novo strategies for generating AMPs have been reported in the literature. For instance, Lee et al. recently published a study wherein they synthesized short peptides (5-11 residues) composed of only three amino acids: Lys, Leu and Trp [36]. Their results support the notion that the antimicrobial activity of a peptide is related to peptide amphipathicity and helicity as well as to the location of the Trp residues. However, their most potent peptide (LKWLLKWLL-NH_2_) also displayed significant hemolytic activity, indicating that further refinement of these peptide sequences is required to optimize their use as antimicrobials.

This study demonstrates that de novo generation of AMPs is still not a trivial endeavour and our current understanding of AMPs is insufficient to predict the antimicrobial potency of novel peptide sequences. Be that as it may, the methodology described here was successful in generating an AMP (gp41w-FKA) with comparatively low cytotoxicity from an intrinsically inactive membrane-associated peptide sequence. With further modifications, the gp41w-FKA peptide can be taken as a starting point to create a potent AMP.

## Experimental

### Peptide synthesis and chemical reagents

Gp41w, gp41w-4R, gp41w-KA, gp41w-FKA were synthesized by Anaspec Inc. (Fremont, CA) to >95% purity. Peptide concentrations were determined from absorbance measurements at 280 nm and theoretical extinction coefficients obtained from Protparam on the EXPASY web server [37]. Powdered dodecylphosphocholine (DPC) and stock chloroform solutions of 1,2-dipalmitoyl-*sn*-glycero-3-phosphocholine (DPPC), 1,2-dipalmitoyl-*sn*-glycero-3-phospho-(1'-*rac*-glycerol) (DPPG), egg-derived L-α-phosphatidylcholine (ePC), egg-derived L-α-phosphatidylethanolamine (ePE), egg-derived L-α-phosphatidylglycerol (ePG) and cholesterol were purchased from Avanti Polar Lipids, Inc. (Alabaster, AL). Deuterated methanol used in the NMR samples was obtained from Cambridge Isotopes Laboratories, Inc. (Andover, MA). Deuterated chloroform (CDCl_3_) was obtained from Norell, Inc. (Landisville, NJ). All other chemicals were purchased from Sigma Aldrich (St. Louis, MO).

### Biological activity

Minimum inhibitory concentrations (MIC) and minimum bactericidal concentrations (MBC) were determined for *E. coli* and *S. aureus* as described previously [38]. Hemolytic activity was also determined by using red blood cells from healthy volunteers, as described previously [38].

### Large unilamellar vesicle preparation

Lipid mixtures consisting of 1:1 weight ratios of ePC, ePG and ePE were prepared from stock lipid solutions in chloroform. The organic solvent was evaporated under a stream of nitrogen gas and the resulting lipid cake was placed under vacuum for ~2 h then stored at −20 °C until ready for use. Large unilamellar vesicles (LUVs) were prepared by warming the lipid film to room temperature followed by resuspension in buffer (10 mM Tris, 150 mM NaCl, 1 mM EDTA, pH 7.4) with vigorous vortexing. The lipid suspension was subjected to five rounds of freezing and thawing in liquid nitrogen followed by 15 passes through two 100 nm polycarbonate filters using the Avanti Mini-Extruder apparatus. The lipid concentration in the LUV samples was determined by measuring the phosphate concentration using the assay described by Ames [39].

### Tryptophan emission fluorescence

Intrinsic tryptophan fluorescence of the peptides was measured on a Cary Eclipse Fluorimeter (Agilent Technologies, Victoria, Australia) equipped with a temperature control unit set at 25 °C. Emission spectra were recorded between 300 and 400 nm with an excitation wavelength of 295 nm. Excitation and emission slits were kept at 5 and 10 nm, respectively, for all the peptides except for gp41w-FKA, which has a single Trp residue, and for which both slit widths were maintained at 10 nm. The concentration of SDS and DPC in the fluorescence samples was 25 mM, while the lipid concentration in the LUV samples was 30 μM. Acrylamide titrations were performed to determine *K*_sv_ [40] by adding five aliquots of a 4 M acrylamide solution to the sample and recording an emission spectra using the parameters described above. *K*_sv_ was calculated by using the formula *F*_0_/*F* = 1 + *K*_sv_/[Q], Where *F*_0_ is the initial fluorescence and *F* is the fluorescence intensity after the addition of soluble quencher (Q).

### Calcein leakage

Calcein leakage was performed according to established protocols [41]. Melittin, the principal component of bee venom and a known lytic peptide, was used as a positive control. Calcein leakage was measured for LUVs made from lipid mixtures of ePC:ePG, ePE:ePG or ePC:ePE.

### Differential scanning calorimetry

Lipid films of 0.5 mg pure DPPC or DPPG were prepared as described above and stored at −20 °C until needed. Lipid films and buffer were heated to 55 °C for 15 min and then hydrated with buffer (20 mM phosphate buffer, 130 mM NaCl, pH 7.4) and vortexed vigorously. Following resuspension, stock solutions of peptides in water were added to achieve a final lipid:peptide molar ratio of 10:1. The final lipid concentration in each sample was 0.5 mg/mL. Differential scanning calorimetry was performed on a MicroCal high sensitivity VP-DSC machine (GE Healthcare, Piscataway, NJ). Five cycles of heating between 20 and 60 °C, at a scan rate of 10 °C/h were performed and the peptide–lipid mixture was added as the cells cooled between the first and second scans. Since the peptide was added exogenously to the lipid suspensions, the four remaining heating and cooling cycles allowed the peptide to equilibrate amongst all the lipid molecules. In all cases, the final scan was virtually identical to the previous scan, indicating that equilibrium had been reached.

### Circular dichroism spectroscopy

CD spectra were collected at room temperature on a Jasco J815 spectropolarimeter by using a 0.1 cm path length cuvette. Far-UV spectra of 50 μM peptide solutions were collected between 190–260 nm with a 0.5 nm step resolution, a 200 nm/min scan speed, a response time of 0.5 s and a bandwidth of 1 nm. CD spectra were acquired for the peptide in buffer (25 mM sodium phosphate, pH 7.4) and in buffered solutions containing 25 mM SDS, 25 mM DPC and 50% 2,2,2-trifluoroethanol. Spectra were also recorded for peptides in the presence of LUVs composed of ePC:ePG, ePE:ePG, and ePC:ePE. The lipid concentration in each of the LUV samples was maintained at 0.5 mM. Each spectra represents the average of 10 scans, and a blank spectra, lacking peptide, was subtracted in the final analysis. Data was converted to mean residue ellipticity according to Wallace and Janes [42].

### NMR structure determination

The solution structures of gp41w-4R, gp41w-KA and gp41w-FKA were determined in the 4:4:1 cosolvent mixture of CDCl_3_:methanol-*d*_3_:H_2_O previously used by our group to study the NMR solution structures of other AMPs [19–20,43]. NMR samples were prepared by dissolving 1–2 mg of lyophilized peptide powder in 500 μL of the miscible cosolvent solution, and then the samples were flame sealed in an NMR tube to avoid evaporation of the organic solvents. Two-dimensional NOESY, TOCSY and COSY spectra were collected at 298 K on a Bruker Avance 700 MHz spectrometer. Mixing times in the NOESY and TOCSY experiments were 100 ms and 120 ms, respectively. Spectra were acquired with 4096 × 600 data points in the F1 and F2 dimensions at a spectral width of 8992.806 Hz. Water suppression was achieved by using excitation sculpting [44].

All of the spectra were processed with NMRPipe [45]. The 2D data was zero-filled once in each dimension and Fourier transformed with a shifted sine-bell function. Spectra were analyzed with NMRView [46] and chemical shifts were assigned according to Wuthrich [47]. Starting structures of the gp41 peptides were generated with CNS [48] and broad dihedral restraints were placed on the backbone *phi* and *psi* angles to maintain these angles in allowable regions of the Ramachandran plot [49]. Solution structures were calculated based on the NOESY-derived distance restraints by using the simulated annealing protocol in ARIA1.2 [50]. Nine iterations of the simulated annealing protocol were performed with 20 structures generated in the first seven iterations followed by 40 and 100 in the final two iterations. The 20 lowest energy structures from the final iteration were analysed with Procheck [51] and visualized with MOLMOL [52].

## Supporting Information

File 1 NMR spectra and peptide connectivity analysis based on the observed NOEs and structural statistics for the calculated NMR structures.
