# Supplementary material for: Design of a novel tryptophan-rich membrane-active antimicrobial peptide from the membrane-proximal region of the HIV glycoprotein, gp41
Source: Beilstein J Org Chem. 2012 Jul 24;8:1172–84. doi: 10.3762/bjoc.8.130 (PMC3458735; doi:10.3762/bjoc.8.130)
Supplement: File 1 — NMR spectra and peptide connectivity analysis based on the observed NOEs and structural statistics for the calculated NMR structures. [file Beilstein_J_Org_Chem-08-1172-s001.pdf]

Supporting Information

for

Design of a novel tryptophan-rich membrane-active antimicrobial peptide from the membrane-proximal region of the HIV glycoprotein, gp41

Evan F. Haney, Leonard T. Nguyen, David J. Schibli[§] and Hans J. Vogel[*,$]

Address: Department of Biological Sciences, University of Calgary, 2500 University Drive NW, Calgary, Alberta, Canada, T2N 1N4

Email: Hans J. Vogel* - vogel@ucalgary.ca

*Corresponding author

[§]Current address: Ontario Genomics Institute, MaRS Centre, Heritage Building, 101 College Street, Suite HL50, Toronto, Ontario, Canada, M5G 1L7

[$]Phone: 1-403-220-6006

NMR spectra and peptide connectivity analysis based on the observed NOEs and structural statistics for the calculated NMR structures

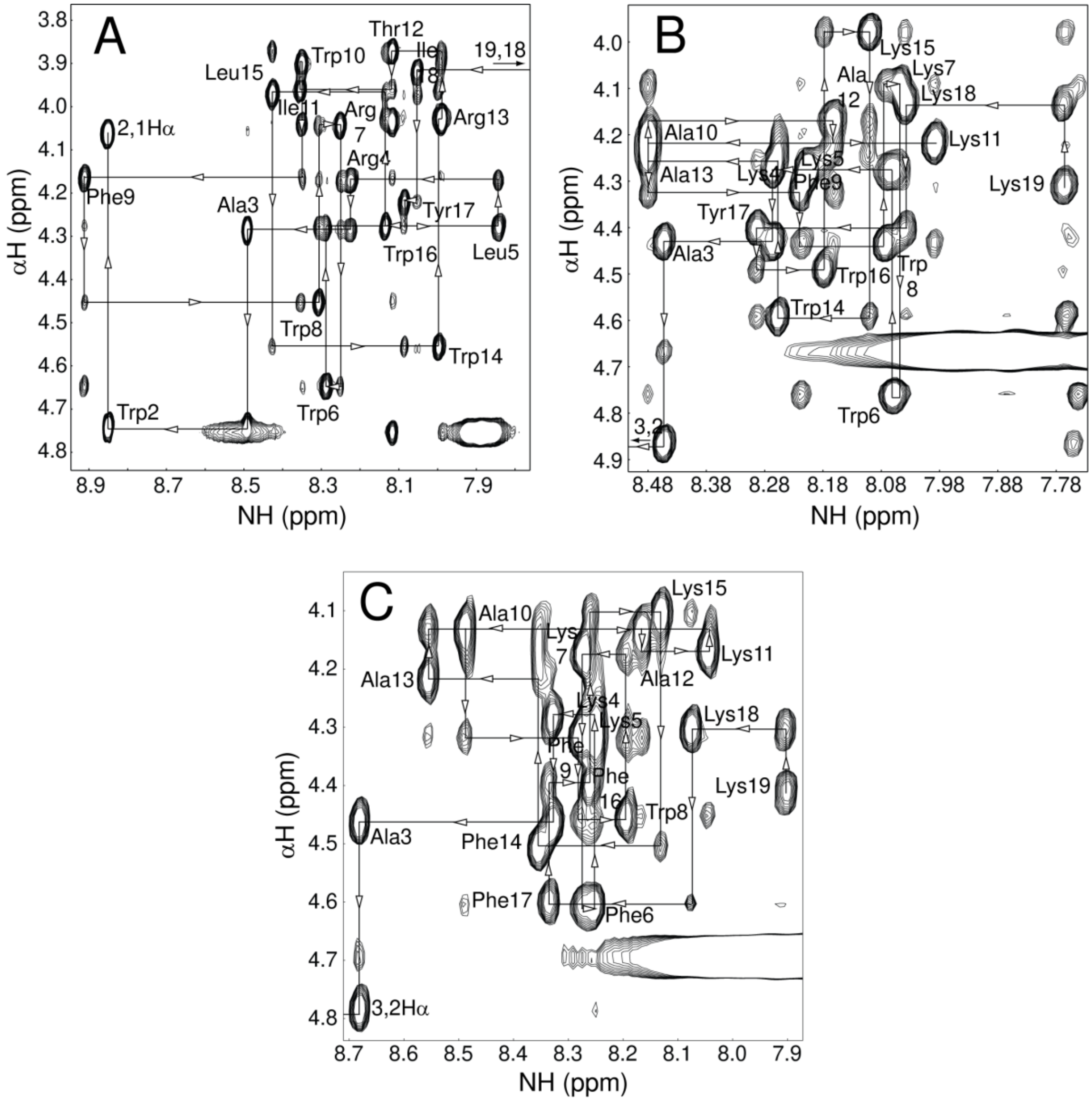

Figure S1: Representative regions of the 2D ^{1}H NOESY spectra of gp41w-4R (A), gp41w-KA (B) and gp41w-FKA (C) acquired on a Bruker 700 MHz spectrometer. The NH-αH fingerprint regions are shown with the sequential connectivity between NH_i to αH_{i-1} indicated.

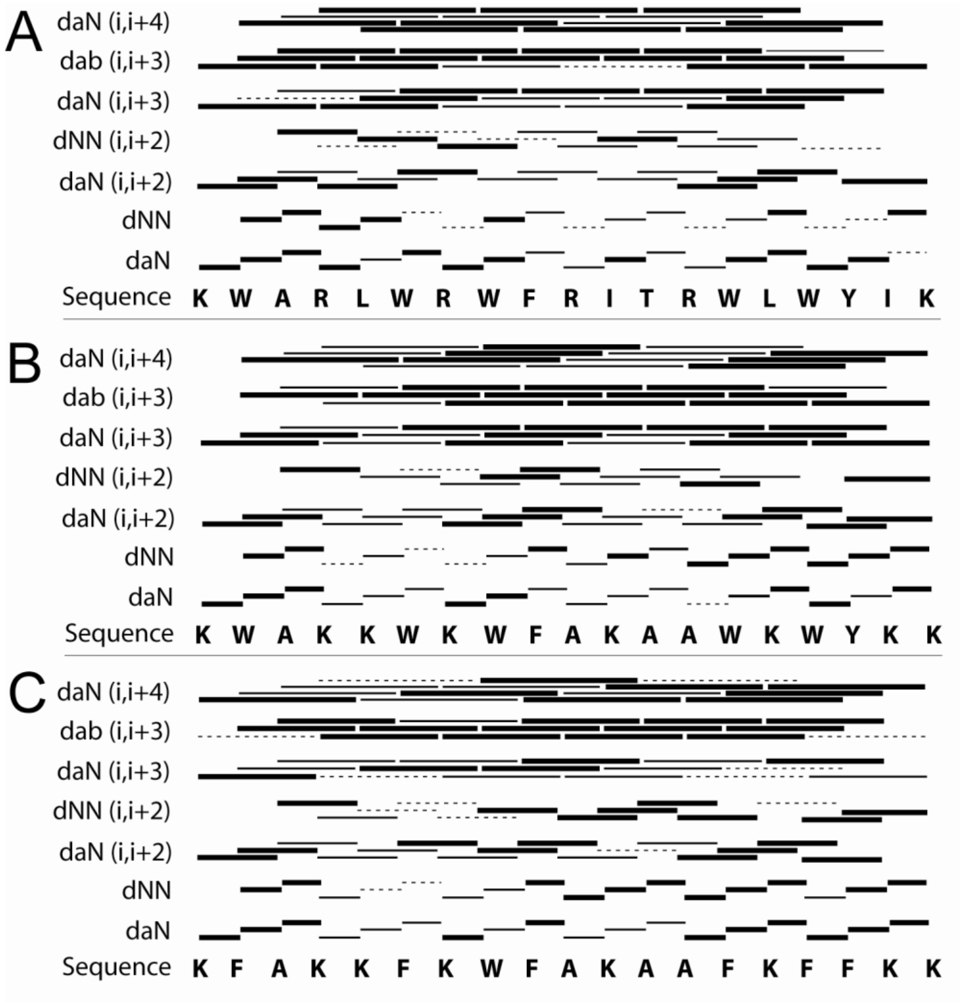

Figure S2: Intramolecular NOE contacts observed in the 2D [1] NOESY spectra of gp41w-4R (A), gp41w-KA (B) and gp41w-FKA (C) in the miscible cosolvent mixture. Unambiguous (thick lines) and ambiguous (thin lines) assignments were used by Aria for the simulated annealing protocol to calculate peptide conformations. Ambiguous peaks that had density in the spectra but were occluded by nearby peaks are also indicated (dashed lines), but these restraints were not included in the structure calculations.

Table S1: Structural statistics for the NMR solution structures of gp41w-4R, gp41w-KA and gp41w-FKA determined in a cosolvent mixture of chloroform, methanol and water.

	gp41w-4R	gp41w-KA	gp41w-FKA
No. of distance restraints			
Unambiguous NOEs	369	444	328
Ambiguous NOEs	24	33	20
Unassigned NOEs	2	1	0
Total NOEs	395	478	348
Dihedral Restraints	18	18	18
RMS deviations among the structures			
Bonds (Å)	1.23×10^{-2} +/− $6.3 \text{ x } 10^{-5}$	1.22×10^{-2} +/− 1.89×10^{-4}	1.18×10^{-2} +/− 6.41×10^{-5}
Angles (degree)	0.688 +/− 1.4×10^{-2}	0.819 +/− 1.06×10^{-2}	0.684 +/− 6.86×10^{-3}
Impropers (degree)	0.187 +/− 9.49×10^{-3}	0.461 +/− 8.58×10^{-3}	0.185 +/− 9.12×10^{-3}
Dihedrals (degree)	21.1 +/− 1.20	20.8 +/− 1.07	17.2 +/− 0.672
Pairwise atom RMSD (Å)			
All atoms	1.618	1.380	1.302
Heavy atoms	1.297	1.087	0.963
Backbone	0.630	0.389	0.426
Ramachandran space (%)[a]			
Most favoured	82.1	76.8	85.3
Additionally favoured	17.9	22.9	14.7
Generously allowed	0.0	0.3	0.0
Disallowed	0.0	0.0	0.0

[a]Calculated by Procheck (42).
